# Sanjin tablets for acute uncomplicated lower urinary tract infection (syndrome of dampness-heat in the lower jiao): protocol for randomized, double-blind, double-dummy, parallel control of positive drug, multicenter clinical trial

**DOI:** 10.1186/s13063-019-3539-5

**Published:** 2019-07-19

**Authors:** Jian Lyu, Yan-ming Xie, Zhan Gao, Jian-wu Shen, Yue-yi Deng, Song-tao Xiang, Wen-xi Gao, Wen-tong Zeng, Chun-he Zhang, Dan-hui Yi, Lian-xin Wang, Zhi-fei Wang

**Affiliations:** 1 Institute of Basic Research in Clinical Medicine, China Academy of Chinese Medical Sciences, Beijing, 100700 People’s Republic of China; 2 XiYuan Hospital, China Academy of Chinese Medical Sciences, Beijing, 100091 People’s Republic of China; 3 Longhua Hospital Shanghai University of Traditional Chinese Medicine, Shanghai, 200032 People’s Republic of China; 4 Guangdong Provincial Hospital of Traditional Chinese Medicine, Guangdong, 510120 People’s Republic of China; 5 Hubei Provincial Hospital of Traditional Chinese Medicine, Hubei, 430061 People’s Republic of China; 6 Affiliated hospital of Chengdu University of Traditional Chinese Medicine, Chengdu, 610075 People’s Republic of China; 7 Yunnan Provincial Hospital of Traditional Chinese Medicine, Yunnan, 650021 People’s Republic of China; 8 School of Statistics, Renmin University of China, Beijing, 100872 People’s Republic of China

**Keywords:** Acute uncomplicated UTI, Syndrome of dampness-heat in the lower jiao, Randomized controlled trial, Clinical trials, Sanjin tablets

## Abstract

**Background:**

Acute uncomplicated lower urinary tract infection (UTI) is one of the most common bacterial infections. Patients usually present with dysuria, urinary urgency, urinary frequency, and suprapubic pain or tenderness. Approximately 150 million people are diagnosed with UTI each year worldwide. The high recurrence rate of lower UTI is a common problem of clinical treatment. The misuse of antibiotics has led to the emergence of a number of resistant bacterial strains. Thus, traditional Chinese medicine is considered as an alternative option for treating acute uncomplicated lower UTI. Thus, this study aims to evaluate the efficacy and safety of Sanjin tablets (SJT) for the treatment of acute uncomplicated lower UTI, explore whether SJT can reduce or substitute the use of antibiotics, and reduce the recurrence rate in the treatment of acute uncomplicated lower UTI.

**Methods/design:**

In this study, a randomized, double-blind, double-dummy, parallel control of positive drug, multicenter clinical study will be established. A total of 252 patients with acute uncomplicated lower UTI (syndrome of dampness-heat in the lower jiao) will be randomly allocated in the ratio of 1:1:1 to three groups: experimental group; control group 1; and control group 2. The experimental group receives Sanjin tablets plus levofloxacin tablets (LT) placebo; the control group 1 receives LT plus SJT placebo; and the control group 2 receives SJT plus LT on the first five days, SJT plus LT placebo on the last two days. Each group will be treated for seven days and followed-up 1–2 times. The primary outcome measures of effective rate and recurrence rate are symptoms. Secondary outcome measures of effective rate and recurrence rate are the urine leukocytes, bacteriology examination, and safety assessment. Outcomes will be assessed at baseline and after treatment.

**Discussion:**

This study protocol will provide the research data of efficacy and safety of SJT for the treatment of acute uncomplicated lower UTI. The first aim is to determine whether Sanjin tablets can reduce the use of antibiotics; the second aim is to determine whether Sanjin tablets can substitute the use of antibiotics. The recurrence rate will be assessed after cured to determine whether SJT can reduce the recurrence rate. The results of this study will improve the rational use of drugs, especially the rational application of antibiotics. It will also enable safety evaluation from laboratory indices and adverse events, which will provide reliable evidence for clinical treatment.

**Trial registration:**

ClinicalTrials.gov, NCT03658291. Registered on 4 September 2018.

**Electronic supplementary material:**

The online version of this article (10.1186/s13063-019-3539-5) contains supplementary material, which is available to authorized users.

## Background

Acute uncomplicated lower urinary tract infection (UTI) is one of the most common bacterial infections. Patients usually present with dysuria, urinary urgency, urinary frequency, and suprapubic pain/tenderness [1]. In the United States, there are > 7 million outpatients with UTIs and about 1 million inpatients each year [2]. Approximately 150 million people are diagnosed with UTI each year worldwide [3]. UTI, which can cause shock and death, ranks third among all diseases that die from infection [4]. Gram-negative bacteria, especially Enterobacteriaceae, is the common cause of both community-acquired and hospital-acquired UTIs [3, 5]. Many Chinese medicines that have a heat-clearing and detoxicating function have been proven to have the bacteriostasis on pathogenic microorganisms, which can inhibit or destroy the formation of toxic substances [6, 7]. In the antibacterial test against mice, it was found that Sanjin tablets (SJT) have strong bacteriostasis activity [8].

SJT have the effects of clearing heat, detoxicating, eliminating dampness, and treating stranguria [9]. It is used to treat urinal urgency, frequency, and burning caused by syndromes of dampness-heat in the lower jiao, which coincides with the symptoms of acute uncomplicated lower UTI. SJT are composed of five kinds of Chinese herbal medicines: Jinyinggen (Root of Cherokee Rose), Baqia (Chinaroot Greenbrier Rhizome), Yangkaikou (Fruit of Fiverleaf Akebia), Jinshateng (Lygodii Herba) and Jixuecao (Asiatic Pennywort Herb), all of which are recorded in the Chinese Pharmacopoeia (V.2015). Jinyinggen (Root of Cherokee Rose) is acerb and neutral in nature; it can control nocturnal emission and astringent intestine. Baqia (Chinaroot Greenbrier Rhizome) is cosin and neutral in nature; it can remove rheumatism, promote blood circulation, detoxicate, relieve convulsion, and calm endogenous wind. Jinshateng (Lygodii Herba) is slightly sweet and cold-natured; it can clear heat, detoxicate, and remove dampness. The above three herbs are monarch drugs in a prescription, which have a mutual promotion effect on the function of clearing heat, removing dampness, and inducing diuresis for treating stranguria. The above three medicines, supplemented with Yangkaikou (Fruit of Fiverleaf Akebia) and Jixuecao (Asiatic Pennywort Herb), can enhance the effects of removing dampness and treating stranguria [10, 11].

SJT are a completely natural preparation with significant efficacy in treating UTI [12]. Previous clinical studies have indicated that SJT can reduce the symptoms of chronic UTI and number of acute attacks and can reduce the secretory level of urinary sIL-2R, IL-6, IL-8 in patients with chronic nephropyelitis [8]. Observation by electron microscope found that SJT can make *Escherichia coli* flagella drop without any danger. A meta-analysis has demonstrated that the current evidence is insufficient to support the efficacy and safety of SJT for acute uncomplicated lower UTI due to lack of high-quality randomized controlled trials (RCT) [13]. The high recurrence rate of lower UTI is a common problem of clinical treatment [14]. The misuse of antibiotics has led to the emergence of a number of resistant bacterial strains [15]. Unfortunately, the efforts to produce new antibiotics have not been sufficient to cope with the emergence of these new antibiotic-resistant (AR) strains [16]. Therefore, the objective of our study is to evaluate effectiveness, safety, and recurrence rate of SJT and explore whether SJT can reduce or substitute the use of antibiotics for acute uncomplicated lower UTI by a rigorous RCT.

## Method/design

### Objective and design

**Fig. 1:**
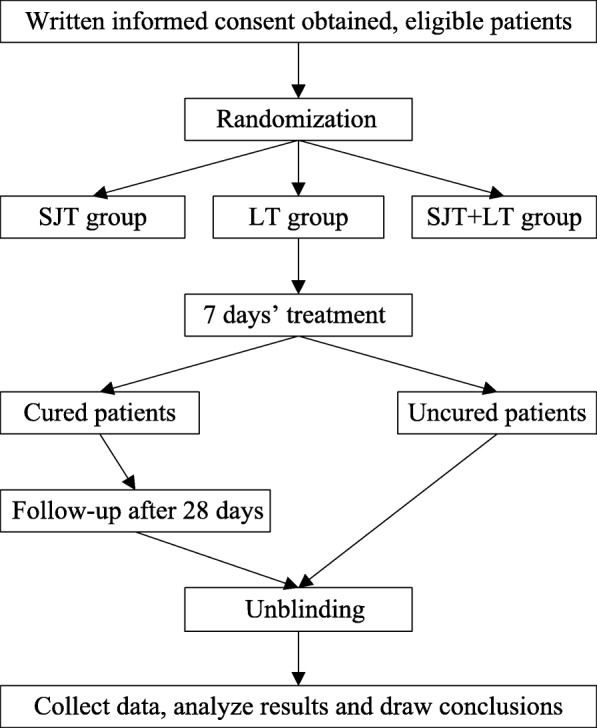
Flow diagram. SJT Sanjin tablets, LT levofloxacin tablets

This is a randomized, double-blind, double-dummy, parallel control of positive drug, multicentre clinical study. The study will be conducted at: the XiYuan Hospital, China Academy of Chinese Medical Sciences; the Longhua Hospital Shanghai University of Traditional Chinese Medicine; the Guangdong Provincial Hospital of Traditional Chinese Medicine; the Hubei Hospital of Traditional Chinese Medicine; the Affiliated hospital of Chengdu University of Traditional Chinese Medicine; and the Yunnan Provincial Hospital of Traditional Chinese Medicine. Ethical clearance for the trial was obtained from ethics committee of the six hospitals. Patients with acute uncomplicated lower UTI will undergo a standardized baseline evaluation before treatment, comprising detailed history taking, physical examination, and laboratory testing. All included patients are randomly divided into three groups: experimental group; control group 1; and control group 2. The experimental group receives SJT plus levofloxacin tablets (LT) placebo; control group 1 receives LT plus SJT placebo; and control group 2 receives SJT plus LT on the first five days, SJT plus LT placebo on the last two days. After seven days of treatment, the efficacy and safety of three groups will be evaluated. The first aim is to determine whether SJT can reduce the use of antibiotics (control group 2 versus control group 1, non-inferiority test); the second aim is to determine whether SJT can substitute the use of antibiotics (experimental group versus control group 1, non-inferiority test). The recurrence rate will be assessed at the 28-day follow-up after being cured to determine whether SJT can reduce the recurrence rate. The trial is conducted in accordance with the World Medical Association Declaration of Helsinki and Good Clinical Practice of Pharmaceutical Products [17, 18]. After a full explanation by the clinicians, written informed consent will be obtained from the participants before intervention [19, 20]. Strict data management and quality control will be conducted in this trial [21, 22]. This trial was registered in the Clinical Trials USA registry (registration No. NCT03658291) on 4 September 2018 and will be carried out from January 2019 to December 2019. The study design is shown in Fig. 1. The protocol follows the recommendations of the SPIRIT initiative (see Additional file 1) [23] and the trial results will be reported according to the latest version of the CONSORT statement [24].

## Patient and public involvement

Patients were not involved in the research question, design, conduct, outcome measures, and data analysis of the study. Only medically trained clinicians carry out the patient recruitment and management in the study. The clinicians will describe the purpose, burden of the intervention, procedure, and potential risks of this trial to the participants themselves or their designated representative before the recruitment. We will disseminate the results of this study to participants through patient organizations and open lectures.

### Sample size

The effective rate will be taken as the main outcome measure of the clinical trial. According to a previous study, it showed that the effective rate of SJT was 77.14% in the treatment of simple bacterial lower urinary tract infection [25]. On the basis of a 0.9 power to detect a significant difference (α = 0.01, two-sided), 76 participants will be required for each group. 228 participants will be required for the three groups in a 1:1:1 ratio. Considering a 20% drop-out rate, we plan to include 252 patients in the whole trial.

### Participants and recruitment

#### Diagnostic criteria of western medicine and traditional Chinese medicine

The western medicine diagnostic criteria of acute uncomplicated lower UTI will refer to the *Guidelines for the diagnosis and treatment of urological diseases in China* (2014 ed) [26]. The traditional Chinese medicine (TCM) diagnostic criteria of acute uncomplicated lower UTI will refer to the *Clinic terminology of traditional Chinese medical diagnosis and treatment-syndromes*, *Internal Medicine of TCM* [27–29]. According to the characteristics and functions of SJT, it is proposed to select the syndrome of dampness-heat in the lower jiao as the syndrome of TCM in this trial.

#### Inclusion criteria

Inclusion criteria are as follows: (i) age > 18 and < 50 years; (ii) meet the western diagnostic criterion of acute uncomplicated lower UTI [26] that the disease duration does not exceed 72 h; (iii) bacterial culture result will be sensitive to levofloxacin (bacterial culture and clinical treatment will be conducted at the same time); (iv) the syndrome differentiation of TCM is syndrome of dampness-heat in the lower jiao; (v) did not receive antibiotic treatment within 48 h before being selected; (vi) not pregnant or lactating; and (vii) sign the written informed consent form for the clinical trial.

#### Exclusion criteria

Exclusion criteria are as follows: (i) allergic to the test drug or quinolones; (ii) there was a history of bacterial culture that was not sensitive to levofloxacin; (iii) diagnosed as complicated UTI; (iv) patients with urinary calculi or obstruction, urinary tuberculosis, renal papillary necrosis, perinephric abscess, or neurogenic bladder; (v) combined with vaginitis symptoms, genital ulcers, or gonorrhea; (vi) combined with severe cardiopulmonary disease, liver and kidney disease, advanced tumor, and other serious or progressive disease; (vii) have a neurological or mental illness and not able to cooperate; (viii) use of other antibacterial drugs in combination; (ix) pregnant, lactating, planning to become pregnant women; (x) taking part in another clinical trial within three months before being selected.

#### Exit criteria

Patients will leave the trial when one of the following criteria is met: (i) incorrectly included; (ii) are poorly compliant or pregnant; (iii) no medication or any follow-up records; (iv) occurrence of allergic reactions or serious adverse events (AE); (v) participants have other complications or special physiological changes during the trial; (vi) patients have been treated with other medicines during the trial; and (vii) not alleviated or the symptoms are aggravated. Participants may withdraw from the study at any time for any reason.

### Blinding

This is a double-blind (patients and clinicians are blinded) and double-dummy study [30]. A two-stage blind design was used. The first stage was group A, group B, and group C; the second stage was randomization to the experimental group, control group 1, and control group 2 according to SAS software, without correspondence to group A, group B, and group C. Treatment assignments will not be revealed until the whole process is complete. If patients have severe AEs, clinicians will log on to the central platform to unblind the patients as an emergency and provide relevant treatment. Once the blinding is broken, the patient with this number will be withdrawn from the trial and the clinicians will record the reasons in the case report form (CRF). To achieve blinding, all three groups will use the same kind of packaging to encase the drug or placebo. Size, color, shape, taste, smell, and packaging of the placebo are made identical to that of the corresponding medicine by adding artificial pigment.

## Randomization and allocation concealment

Randomization will be used for patient allocation [31]. The randomization will be performed by an independent statistician. The random numbers are divided into three groups sequentially: experimental group; control group 1; and control group 2. SAS 9.1.3 statistical software PROC PLAN procedure statements will be used, the seed number given, and randomized grouping tables will be generated for the 252 patients receiving treatment. On-site drug blinding will be carried out, and emergency unblinding procedures will be created. Blinded materials will be kept by a full-time investigator who will not participate in any part of the trial. During the trial, the investigator will be able to obtain the randomized number and drug number of each patient from the designated central randomized platform.

### Interventions

All researchers are clinical doctors and receive standardized training for the diagnostic interview before the start of the trial. LT are licensed for UTI with proven curative effect and safety [32]. Therefore, levofloxacin had been selected as the active control medicine. Participants in the experimental group will receive SJT plus LT placebo orally four times a day for seven days. Participants in control group 1 will take LT plus SJT placebo four times a day for seven days. Participants in control group 2 will take SJT and LT four times a day for the first five days and SJT plus LT placebo four times a day for the last two days. Patient visits are required after seven days of treatment. This is a placebo-controlled study; all participants receive the same number of pills with varying proportions of active drug and placebo to ensure that patients and clinicians are not aware of the allocated treatment aim. The SJT placebo is composed of 55% microcrystalline cellulose, 41% starch, 2% caramel, 1.5% silicon dioxide, and 0.5% talcum powder. The LT placebo is made of 56% microcrystalline cellulose, 42% starch, 1.5% silicon dioxide, and 0.5% talcum powder. The SJT and LT placebos are similar to the SJT and LT in size, color, shape, taste, smell, and packaging. SJT and its placebo and the LT placebo are produced by Guilin Sanjin Pharmaceutical Co., Ltd. and can be preserved for two years. LT is provided by First Sankyo Pharmaceutical (Beijing) Co., Ltd. at a dosage of 100 mg and can be preserved for two years. The recommended dosage of LT is 2–3 times a day, one pill at a time; hence, in order to meet the requirements of the blinding, the administration method of this medicine has been designed as described in Table 1.

**Table 1:** Administration methods in the experimental and control groups

Time	Experimental group	Control group 1	Control group 2	
			First 5 days	Last 2 days
08:00	3 pills of SJT, 1 pill of LT placebo	3 pills of SJT placebo, 1 pill of LT	3 pills of SJT, 1 pill of LT	3 pills of SJT, 1 pill of LT placebo
12:00	3 pills of SJT	3 pills of SJT placebo	3 pills of SJT	3 pills of SJT
17:00	3 pills of SJT, 1 pill of LT placebo	3 pills of SJT placebo, 1 pill of LT	3 pills of SJT, 1 pill of LT	3 pills of SJT, 1 pill of LT placebo
22:00	3 pills of SJT	3 pills of SJT placebo	3 pills of SJT	3 pills of SJT

## Follow-up

**Fig. 2:**
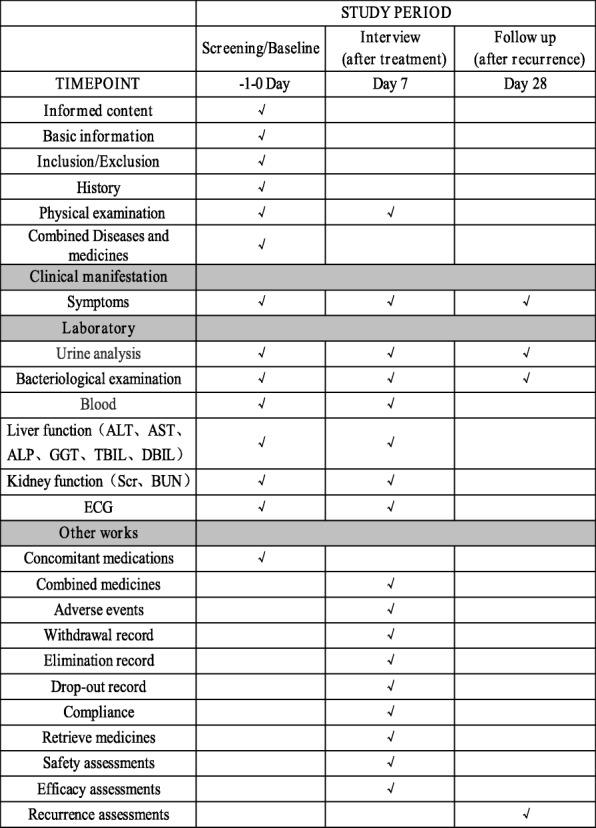
Standard Protocol Items: Recommendation for Interventional Trials (SPIRIT) figure

All included patients will be re-evaluated after seven days of treatment follow-ups as an outpatient to assess the efficacy and safety. The patients who were cured will receive the second follow-up after 28 days to assess the recurrence rate. Patients whose symptoms worsened will receive a supply of relevant medicine and a written withdrawal schedule. Assessment of study endpoints and duration of follow-up is shown in Fig. 2.

## Outcome measures

### Effective rate

The effective rate will be evaluated from three dimensions: symptoms; urine leukocytes; and bacteriological examination.

#### Primary outcome measure

The symptoms of acute uncomplicated lower UTI will be assessed. The assessment tools for symptoms of acute uncomplicated lower UTI are three participant self-rating scales (see Additional file 2): criteria sheet for evaluating the severity of urinary system infection (CSESUSI; mild: total score ≤ 8, moderate: total score 9–15, severe: total score ≥ 16), if the severity of the disease decreases from severe to moderate, or from moderate to mild, the symptoms are improved; symptoms score sheet of TCM (SSST; cure: total score = 0, effective: total score 0–6, invalid: total score ≥ 6); overactive bladder symptom score (OABSS; if the urgent urination score > 2 and total score > 3, OAB will be diagnosed; mild: total score ≤ 5, moderate: total score 6–11, severe: total score ≥ 12). The CSESUSI will use a scoring method to record the changes of symptoms and signs before and after treatment, such as shivering, fever, dysuria, urinal pain, urinary urgency, urinary frequency, lower abdomen distending pain, lumbago, and percussion tenderness over kidney region. The SSST uses a scoring method to record the changes of symptoms, tongue, and pulse in TCM before and after treatment. The OABSS has been found to be reliable and valid and highly responsive to treatment-related fluctuations in overactive bladder symptoms. Repeating the test after an acceptable treatment interval is an effective way to assess the efficacy of a particular drug regimen on OAB symptoms [33, 34]. If the symptoms disappeared after seven days of treatment, it indicates that the patient is cured. If these symptoms partially disappeared after seven days of treatment, it indicates that the treatment is effective but not cured. If there was no obvious remission and the symptoms of infection persisted after seven days of treatment, it indicates that the treatment is invalid.

#### Secondary outcome measure

(i) The urine leukocytes of 252 participants will be assessed. If the value of urine leukocytes is returned to normal after seven days of treatment, it indicates the patient is cured. If the value of urine leukocytes is reduced but not returned to normal after seven days of treatment, it indicates that the treatment is effective. If the value of urine leukocytes is still higher than the normal value after seven days, it indicates the treatment is invalid.
(ii) The bacteriological examination of 252 participants will be assessed. If the original infected part of the specimen did not regenerate the original infected pathogen after seven days of treatment, it indicates the original bacteria is cleared. If the original pathogen was still cultured in the culture of the specimen from the original infection position, it indicates the original bacteria is not cleared.

#### Criteria of comprehensive efficacy

(i) Criteria of cure: the evaluation of clinical symptoms reached the level of cure after seven days of treatment; the value of urine leukocytes returned to the normal value; the original infected bacteria was cleared after seven days of treatment. All three criteria must be met.
(ii) Criteria of effective treatment: the clinical symptoms were alleviated but did not reach the level of cure after seven days of treatment; the value of urine leukocytes was reduced but not returned to normal after seven days of treatment. Either of these criteria must be met.
(iii) Criteria of invalid treatment: the clinical symptoms were not alleviated after seven days of treatment; the value of urine leukocytes was still higher than normal after seven days of treatment; the original infected bacteria were still cultured from the original infection position after seven days of treatment. All three criteria must be met.

### Recurrence rate

Patients who were cured will attend the second follow-up after 28 days to assess the recurrence rate. Recurrence criteria must include item (i) and either (ii) or (iii) at the same time, or item (i) alone.

#### Primary outcome measure

(i) The recurrence rate of cured participants will be assessed by the symptoms of acute uncomplicated lower UTI. The assessment tools of renewed symptoms will also use the three participant self-rating scales (see Additional file 2): CSESUSI; SSST; and OABSS. If the symptoms of cured patients reappeared in the fourth week after the end of medication, it indicates the participant has relapsed.

#### Secondary outcome measures

(ii) The recurrence rate of cured participants will be assessed by urine leukocytes. If the urine leukocyte value of cured patients increased again in the fourth week after the end of medication, it indicates the participant has relapsed.
(iii) The recurrence rate of cured participants will be assessed by bacteriological examination. If the urine culture of cured patients indicated that the original urinary tract pathogen is positive again in the fourth week after the end of medication, it indicates the participant has relapsed.

### Safety assessments

Safety measurements included laboratory indices and AEs. All patients will undergo the following laboratory examination before enrollment and at the end of the clinical trial. Changes in laboratory indices before and after the clinical trial will be compared to conducting a safety analysis. Laboratory indices are: (i) routine blood and urine testing; (ii) liver function (AST, ALT, TBIL, DBIL, γ-GT, ALP) and kidney function (Scr, BUN); and (iii) ECG. The occurrence of any AEs in trial participants, such as subjective discomfort of patients and abnormal laboratory results, will be recorded in the CRF during the whole trial process. We will withdraw patients who have severe AEs, as it will be unsafe for them to continue the trial. Meanwhile, we will give them relevant medical care and follow them up until the reaction has terminated.

## Informed consent

Before enrolling patients in the trial, the investigating doctor will completely and comprehensively describe the purpose, procedure, and potential risks of this trial in writing to the patient themselves or their designated representative. Patients will be informed that they have the right to withdraw from the trial at any time. Each patient must provide written informed consent before participating in this study; this consent will be kept in the study file.

## Quality control

Quality assessment will be conducted in the following aspects in this trial: the progress of the trial; the qualifications of the investigators; the mastery of the program; the authenticity, accuracy, and completeness of the CRF; archival preservation; program implementation; AEs; drug preservation and storage; written informed consent; participant compliance; and laboratory examination data. In particular, the authenticity and accuracy of the CRF, program implementation, and determination of AEs will be strictly inspected.

### Data management

The patients in this trial will be recruited patients. Therefore, the original data will include the CRF, patient log card, original laboratory examination, and written informed consent. The inspector will regularly visit all centers to conduct a data quality inspection. The authenticity and accuracy of data will be checked by original laboratory comparison, telephone follow-up with patients, and examination of the integrity, timeliness, and normalization of data. The paper form of the data will be collected after approval inspection. The researcher who is responsible for data entry will build an EpiData-based database with double-recorded data entry by two people and consistency testing will be carried out to ensure the accuracy of data.

### Data analysis

Data analysis will be performed by professional statisticians using SPSS 22.0. Three datasets will be conducted: intention-to-treat; per-protocol set; and safety dataset. The intention-to-treat refers to the patients who have been randomized; an intentional analysis will be conducted for curative effect. The per-protocol set refers to all cases that do not violate the protocol and complete the trial; per-protocol set analysis will be conducted for curative effect. The safety dataset refers to the randomized cases that have taken a tested drug at least once with safety evaluation data after treatment. We use mean ± standard deviation (SD) for continuous variables and percentages for categorical variables. In measured indices, the independent t-test will be used for hypothesis testing of the normal variables, while the Wilcoxon rank sum test will be used for hypothesis testing of the skewed variables. The χ^2^ test will be used for hypothesis testing of the counted indices. The statistical analyses will use the two-sided hypothesis test. *P* ≤ 0.05 will be set as the significant level.

### Ethics and dissemination

The protocol has been approved by: the Ethics Committee of XiYuan Hospital, China Academy of Chinese Medical Sciences (identifier 2018XLA03l-3); Ethics Committee of Longhua Hospital Shanghai University of Traditional Chinese Medicine (identifier 2018LCSY034); the Ethics Committee of Guangdong Provincial Hospital of Traditional Chinese Medicine (identifier BF2018–083-01); the Ethics Committee of Hubei Hospital of Traditional Chinese Medicine (identifier SL2018-C19–01); the Ethics Committee of the Affiliated hospital of Chengdu University of Traditional Chinese Medicine (identifier 2018KL-053); and the Ethics Committee of Yunnan Provincial Hospital of Traditional Chinese Medicine (identifier 2018LLZ-014-NO.01). The protocol has been registered in the Clinical Trials USA registry (registration no. NCT03658291) on 4 September 2018 (see Additional file 3). The trial will help to demonstrate if SJT is effective and safe for patients with acute uncomplicated lower UTI. We will publish the results of this study in peer-reviewed journals to ensure widespread dissemination.

## Discussion

This study was designed to evaluate the efficacy, safety, and recurrence rate of SJT for the patients with acute uncomplicated lower UTI. A total of 252 patients were divided into three groups in a randomized, double-blind, double-dummy, parallel control, multicenter clinical trial. To our knowledge, this is the first study protocol to evaluate the recurrence rate and the effects of reducing or substituting antibiotics of SJT for the treatment of acute uncomplicated lower UTI. The first aim of this trial is to determine whether SJT can reduce the use of antibiotics (control group 2 versus control group 1, non-inferiority test); the second aim is to determine whether SJT can substitute the use of antibiotics (experimental group versus control group 1, non-inferiority test). The recurrence rate will be assessed at 28-day follow-up after being cured to determine whether SJT can reduce the recurrence rate. To facilitate high validity and reliability, a strict quality control and high-quality methodology is indispensable. We describe in detail the method of randomization, allocation concealment, blinding, interventions, recruitment, and data collection. The results from this trial may provide evidence on the effectiveness, safety, recurrence rate, and reduce or substitute antibiotics of SJT.

## Strengths and limitations of this study

- This is a well-designed study to assess the efficacy, safety, and recurrence rate of SJT for acute uncomplicated lower UTI in adults.
- The results from this randomized, double-blind, double-dummy, parallel control of positive drug, multicenter clinical trial will provide new evidence of the efficacy and recurrence rate of SJT for acute uncomplicated lower UTT.
- This study will provide evidence of SJT for reducing or substituting the use of antibiotics for acute uncomplicated lower UTI, which can guide rational application of antibiotics.
- This trial will be implemented in six hospitals in Chinese patients; this can strengthen its generalizability.
- One limitation is the results of participant symptoms evaluate scales might have subjective factors.
- Another limitation is the participant symptoms evaluate scales, which might exaggerate the severity of the symptoms of acute uncomplicated lower UTI.

## Trial status

This trial was registered in the Clinical Trials USA registry (registration no. NCT03658291) on 4 September 2018. Recruitment will begin in January 2019 and it is anticipated that enrollment will be completed in December 2019.

## Additional files

Additional file 1: SPIRIT 2013 checklist. This document provides the recommended items to address in a clinical trial protocol and related document. (DOC 167 kb)

Additional file 2: The three participant self-rating scales of symptoms. This document provides the full details of three participant self-rating scales: criteria sheet for evaluating the severity of urinary system infection (CSESUSI); symptoms score sheet of TCM (SSST); OABSS. (DOCX 21 kb)

Additional file 3: Clinical Trials.gov, NCT03658291. This document provides full registration information. (PDF 75 kb)

## Abbreviations

CSESUSI: Criteria sheet for evaluating the severity of urinary system infection
LT: Levofloxacin tablets
NMPA: National Medical Products Administration
OABSS: Overactive Bladder Symptom Score
SJT: Sanjin tablets
SSST: Symptoms score sheet of TCM
TCM: Traditional Chinese medicine
UTI: Urinary tract infection;
