# Supplementary material for: Sanjin tablets for acute uncomplicated lower urinary tract infection (syndrome of dampness-heat in the lower jiao): protocol for randomized, double-blind, double-dummy, parallel control of positive drug, multicenter clinical trial
Source: Trials. 2019 Jul 19;20:446. doi: 10.1186/s13063-019-3539-5 (PMC6642468; doi:10.1186/s13063-019-3539-5)
Supplement: Supplementary file 2 — The three participant self-rating scales of symptoms. This document provides the full details of three participant self-rating scales: criteria sheet for evaluating the severity of urinary system infection (CSESUSI); symptoms score sheet of TCM (SSST); OABSS. (DOCX 21 kb) [file 13063_2019_3539_MOESM2_ESM.docx]

**Sheet 1:**

**Criteria Sheet for Evaluating the Severity of Urinary System Infection (CSESUSI)**

| **Score criteria** | | | **Score** | |
| --- | --- | --- | --- | --- |
| 1. **Symptoms, Signs and Examinations** | | | | |
| Mental state | | 0=normal；1= little bad.；2=worse. | | └─┘ |
| Shiver | | 0=no；1=yes. | | └─┘ |
| Gastrointestinal reaction | | 0=no；1=yes. | | └─┘ |
| Fever | | 0=normal；1=37.1℃≤T≤37.8℃；2=37.8℃＜T≤38.8℃；3=T＞38.8℃. | | └─┘ |
| White blood cell or GRAN Elevated | | 0=no；2=yes. | | └─┘ |
| **B. Symptoms and examinations of UTI** | | | | |
| Urinary frequency | | 0=＜7 times/24h；1=7～10 times/24h；2=11～14 times/24h；3=≥15 times/24h. | | └─┘ |
| Urinary urgency | | 0=no；1=have and can be controlled；2=have and can’t be controlled so long；3=have and can’t be controlled anymore. | | └─┘ |
| urinal pain | | 0=no；1=mild；2=have and can stand；3=obvious and can’t stand. | | └─┘ |
| Lower abdomen distending pain | | 0=no；1=mild；2=obvious. | | └─┘ |
| Lumbago | | 0=no；1=mild；2=can stand；3=obvious and can’t stand. | | └─┘ |
| percussion tenderness over kidney region | | 0=no；1=mild；2=obvious. | | └─┘ |
| Urine microscopic examination | | 0= WBC and RBC are no or ±；  1=WBC or RBC is +；  2=WBC or RBC is++～+++；  3=WBC or RBC is++++。 | | └─┘ |
| Total Scores：└─┴─┘ | | | | |
| Total Scores**= A + B** | | | | |
| Total Scores | □mild：≤8分；□middle：9～15分；□severe：≥16分。 | | | |

**Sheet 2:**

**symptoms score sheet of TCM (SSST)**

| **Primary symptoms** | | **Score** |
| --- | --- | --- |
| Urinary frequency | 0=no; 2=＜10 times / day;  4=10~15 times / day; 6=15times / day. | └─┘ |
| Urinary urgency | 0=no; 2= mild; 4=middle;  6=severe | └─┘ |
| Urethral burning sting | 0=no; 2=mild and can’t affect urination;  4=middle and affect urination;  6=severe and unbearable | └─┘ |
| **Secondary symptoms** | |  |
| Urine yellow and red | 0=no; 1=yellow; 2=urine dark yellow;  3=Urine yellow and red | └─┘ |
| Flatulence and discomfort in the lower abdomen | 0=no; 1=sometime; 2=discontinuous;  3=continue to appear and unbearable | └─┘ |
| Night sweat | 0=no; 2=yes | └─┘ |
| Soreness and weakness of waist and knees | 0=no; 2=yes | └─┘ |
| Lumbago | 0=no; 1= faint pain and sometime;  2=more severe and turning side is unfavorable; 3=hard to bear | └─┘ |
| Fever | 0=no; 2=yes | └─┘ |
| **Total scores** | | └─┴─┘ |
| **Specific description of tongue and pulse** | | |
| Tongue manifestation | □ red tongue  □ yellow greasy moss □ fur white greasy | |
| Pulse condition | □ string and fast □ slippery and fast | |

**Sheet 3:**

**OVERACTIVE BLADDER SYMPTOM SCORE (OABSS)**

| **Questions** | **Frequency** | **Score（“√”）** |
| --- | --- | --- |
| ****1. How many times do you typically urinate from waking in the morning until sleeping at night?**** | 7 or less | 0 |
|  | 8 to 14 | 1 |
|  | 15 or more | 2 |
| ****2. How many times do you typically wake up to urinate from sleeping at** **night until waking in the morning****? | 0 | 0 |
|  | 1 | 1 |
|  | 2 | 2 |
|  | 3 or more | 3 |
| ****3. How often do you have a sudden desire to urinate, which is difficult to defer****? | Not at All | 0 |
|  | less than once a week | 1 |
|  | once a week or more | 2 |
|  | about once a day | 3 |
|  | 2 to 4 times per day | 4 |
|  | 5 times a day or more | 5 |
| ****4. How often do you leak urine, because you cannot defer the sudden desire to urinate****? | Not at All | 0 |
|  | less than once a week | 1 |
|  | once a week or more | 2 |
|  | about once a day | 3 |
|  | 2 to 4 times per day | 4 |
|  | 5 times a day or more | 5 |
