# Supplementary material for: Sanjin tablets for acute uncomplicated lower urinary tract infection (syndrome of dampness-heat in the lower jiao): protocol for randomized, double-blind, double-dummy, parallel control of positive drug, multicenter clinical trial
Source: Trials. 2019 Jul 19;20:446. doi: 10.1186/s13063-019-3539-5 (PMC6642468; doi:10.1186/s13063-019-3539-5)
Supplement: Supplementary file 3 — Clinical Trials.gov, NCT03658291. This document provides full registration information. (PDF 75 kb) [file 13063_2019_3539_MOESM3_ESM.pdf]

ClinicalTrials.gov Protocol Registration and Results System (PRS) Receipt
Release Date: November 22, 2018

ClinicalTrials.gov ID: NCT03658291

Study Identification

Unique Protocol ID: Sanjin tablets

Brief Title: Clinical Study of Sanjin Tablets for the Treatment of Acute Simple Lower Urinary Tract Infection

Official Title: Sanjin Tablets for the Treatment of Acute Simple Lower Urinary Tract Infection and Its Influence on Recurrence Rate: a Randomized, Double Blind, Parallel Control of Positive Drugs, Multi-center Clinical Study

Secondary IDs:

Study Status

Record Verification: November 2018

Overall Status: Not yet recruiting

Study Start: January 1, 2019 [Anticipated]

Primary Completion: June 30, 2019 [Anticipated]

Study Completion: December 31, 2019 [Anticipated]

Sponsor/Collaborators

Sponsor: China Academy of Chinese Medical Sciences

Responsible Party: Principal Investigator
Investigator: Yanming Xie [yanmingxie]
Official Title: Deputy director
Affiliation: China Academy of Chinese Medical Sciences

Collaborators: Xiyuan Hospital of China Academy of Chinese Medical Sciences
Longhua Hospital Shanghai University of Traditional Chinese Medicine
Guangdong Provincial Hospital of Traditional Chinese Medicine
Hubei Hospital of Traditional Chinese Medicine
Chengdu University of Traditional Chinese Medicine
Yunnan Provinical Hospital of Traditional Chinese Medicine

Oversight

U.S. FDA-regulated Drug: No

U.S. FDA-regulated Device: No

U.S. FDA IND/IDE: No

Human Subjects Review: Board Status: Approved
Approval Number: 2018XLA03l-3

Board Name: Ethics Committee of Xi Yuan Hospital of CACMS
Board Affiliation: Xiyuan Hospital CACMS
Phone: 010-62835646
Email: xiyuanlunli@163.com
Address:

No. 1 Xiyuan Playground, Haidian District, Beijing

Data Monitoring: Yes

FDA Regulated Intervention: Yes

Section 801 Clinical Trial: Yes

Study Description

Brief Summary: The purpose of this study is to evaluate the efficacy, safety and immune mechanism of Sanjin tablets for the treatment of acute simple lower urinary tract infection and its influence on recurrence rate.

Detailed Description: In order to evaluating the efficacy, safety and immune mechanism of Sanjin tablets for the treatment of acute simple lower urinary tract infection and its influence on recurrence rate and through research data to guide clinical, improving the rational use of drugs, especially the rational application of antibiotics. In this study, a randomized, double blind, parallel control of positive drugs, multi-center clinical study will be established. According to the relevant regulations of the CFDA, 252 cases need to be registered at least. These cases will be divided into treatment group（Sanjin tablets+ levofloxacin simulants）, control group 1（Sanjin tablets simulants +levofloxacin）and control group 2（Sanjin tablets+ levofloxacin）. Each group will be treated for 7 days and followed up for 2 times. The efficacy indicators of this study were mainly from three dimensions: syndrome, laboratory routine examination and bacteriology examination. The symptom scores and cytokine changes of each group before and after treatment were observed.

Conditions

Conditions: Urinary Tract Infection Lower Acute

Keywords: Sanjin tablets
efficacy and safety
recurrence rate
randomized
double blind
parallel control
multi-center clinical study

Study Design

Study Type: Interventional

Primary Purpose: Treatment

Study Phase: Phase 4

Interventional Study Model: Parallel Assignment

Number of Arms: 3

Masking: Double (Participant, Investigator)

Allocation: Randomized

Enrollment: 252 [Anticipated]

Arms and Interventions

Arms	Assigned Interventions
Experimental: Sanjin tablets group<br>Sanjin tablets+ levofloxacin simulants	Drug: Sanjin tablets<br>Sanjin tablets,3 pills /time, 4 times/ day, for 7 days +levofloxacin simulants,0.1g, po bid, for 7 days.<br>Other Names:<br>• levofloxacin simulants
Placebo Comparator: Levofloxacin group<br>Sanjin tablets simulants +levofloxacin	Drug: Sanjin tablets simulants<br>levofloxacin ,0.1g, po bid, for 7 days+Sanjin tablets simulants,3 pills /time, 4 times/ day, for 7 days.<br>Other Names:<br>• Levofloxacin
Active Comparator: Sanjin tablets+ Levofloxacin group<br>Sanjin tablets+ levofloxacin	Drug: Levofloxacin<br>Sanjin tablets,3 pills /time, 4 times/ day, for 7 days +levofloxacin,0.1g, po bid, for 7 days.<br>Other Names:<br>• Sanjin tablets

Outcome Measures

Primary Outcome Measure:

1. The lower urinary tract infection symptoms of 252 participants will be assessed
   The symptoms mainly includes frequent urination,urgent urination,urinary pain, lower abdominal pain and pain in the kidney area.If these symptoms disappear after 7 days of medication,it indicates the subject is cured.

   [Time Frame: After 7 days of medication]

Secondary Outcome Measure:

2. The urine leukocyte of 252 participants will be assessed
   If the urine leukocyte return to normal value after 7 days of medication,it indicates the subject is cured.

   [Time Frame: After 7 days of medication]
3. The bacteriological examination of 252 participants
   If the original infected part of the specimen did not regenerate the original infected pathogen after 7 days of medication,it indicates the subject is cured.

   [Time Frame: After 7 days of medication]
4. The recurrence rate of subjects who is cured in 252 participants will be assessed by lower urinary tract infection symptoms
   If the lower urinary tract infection symptoms of subjects who is cured appears again in the fourth week after end of medication,it indicates the subject has relapsed.

   [Time Frame: 28 days after the end of treatment]
5. The recurrence rate of subjects who is cured in 252 participants will be assessed by urine leukocyte
   If the urine leukocyte value of subjects who is cured rises again in the fourth week after end of medication,it indicates the subject has relapsed.

   [Time Frame: 28 days after the end of treatment]
6. The recurrence rate of subjects who is cured in 252 participants will be assessed by bacteriological examination

If the urine culture of subjects who is cured indicates that the original urinary tract pathogen is positive again in the fourth week after end of medication,it indicates the subject has relapsed.

[Time Frame: 28 days after the end of treatment]

Eligibility

Minimum Age: 18 Years

Maximum Age: 50 Years

Sex: All

Gender Based:

Accepts Healthy Volunteers: No

Criteria: Inclusion Criteria:

1. Subjects aged 18 to 50 years of age.
2. Meets the western diagnostic criteria of acute simple lower urinary tract infection, the disease duration does not exceed 72 hours.
3. Urine retention in the screening period -1-0 days, bacterial culture results were sensitive to levofloxacin.(Urine retention for bacterial culture and clinical treatment at the same time)
4. The syndrome differentiation of TCM is syndrome of dampness-heat in lower jiao.
5. Did not receive antibiotic treatment within 48 hours Before being selected.
6. The inclusion of those who confirmed not pregnant
7. Those who agree to participate in this clinical trial and sign the informed consent, the process of informed consent meet the relevant provisions of the GCP.

Exclusion Criteria:

1. Those who are allergic to the test drug ingredients or quinolones.
2. In the past, there was a history of bacterial culture that was not sensitive to levofloxacin.
3. Diagnosed as complicated urinary tract infection.
4. Patients with urinary calculi or obstruction, urinary tuberculosis, renal papillary necrosis, perinephric abscess or neurogenic bladder.
5. Combined with vaginitis symptoms, genital ulcers or gonorrhea.
6. Combined with severe cardiopulmonary disease, liver and kidney disease, advanced tumor, blood, central nervous system (such as the history of epilepsy) or other serious or progressive disease.
7. A patient who has a neurological or mental illness and cannot cooperate.
8. Infected persons who must use other antibacterial drugs in combination.
9. Pregnancy, lactating women or recent birth planners.
10. Those who have participated in other clinical trials within 3 months before being selected.

Contacts/Locations

Central Contact Person: Yanming Xie, BA
Telephone: 86-13911112416
Email: ktzu2018@163.com

Central Contact Backup: Lianxin Wang, Doctor
Telephone: 86-13521781839

Email: wlxing@126.com

Study Officials: Yanming Xie, BA
Study Principal Investigator
Institute of Basic Research in Clinical Medicine, China Academy of Chinese Medical Sciences

Locations:

IPDSharing

Plan to Share IPD: No

References

Citations:

Links:

Available IPD/Information:

U.S. National Library of Medicine | U.S. National Institutes of Health | U.S. Department of Health & Human Services
